# 

**Published:** 1963-07

**Authors:** 


### Contents

THE DOCTOR IN THE LAW COURTS . . *Lord Justice Donovan* 65

HOW TO WIN A "HOUSE JOB" . . . . *A. J. G. Dickens* 75

CATARACT . . . . . . . . . *P. Jardine* 84

PAROXYSMAL NOCTURNAL HAEMOGLOBINURIA . . *Clinical-Pathological Conference* 92

OBITUARY . . . . . . . . . . 102

REPORTS FROM SOCIETIES . . . . . . . 104

NOTES AND NEWS . . . . . . . . 105

EXAMINATION RESULTS . . . . . . . 105

APPOINTMENTS . . . . . . . . . 105

